# Standards as applied in reality: a case study on the translation of standards in eHealth evaluation practice

**DOI:** 10.1186/s12911-019-0975-9

**Published:** 2019-11-29

**Authors:** Monika Jurkeviciute

**Affiliations:** Centre for Healthcare Improvement (CHI), Chalmers University of Technology, 41296 Gothenburg, Sweden

**Keywords:** eHealth, Evaluation, Standard, Inter-organizational cooperation, Translation, Factors, Barriers

## Abstract

**Background:**

Application of standards is a way to increase quality in an evaluation study. However, standards are used insufficiently in eHealth evaluation, affecting the generalization of the knowledge generated.

This study aimed to explore how standards are used in a practical setting of an eHealth evaluation, and to identify the factors that can hinder their use.

**Methods:**

The data were collected in a multi-national and interdisciplinary eHealth evaluation study targeted at the elderly people suffering from mild cognitive impairment and mild dementia. The study was carried out in four countries and funded by the European Union. The collected data included meeting minutes (*n* = 8) and e-mail correspondence (*n* = 261) between partners. The chronological sequence of events related to the use of standards was established. Subsequently, the hindering factors related to the use of standards were identified from the sequence.

**Results:**

The use of four standards was described, reflecting a variety of related processes or barriers that emerge during eHealth evaluation. The processes differed based on the type of the standard. Specifically, evaluation frameworks were found to be conceptual standards and they were easy to agree upon, while standardized metrics were more tangible and their use triggered negotiations. Further, the following factors hindered the use of standards in eHealth evaluations: (1) inadequacy of a standard to address a target population or a disease, (2) insufficient resources to use a standard, (3) lack of experience in using a standard, and (4) lack of validation of a standard in a particular location.

**Conclusions:**

Standardization initiatives in eHealth evaluation provide a blueprint for evaluation, but their practical application is problematic. The practical circumstances of an evaluation study can cause deviations in the standards, thus producing heterogeneity in the evaluation methodologies.

## Background

The term eHealth refers to technology-enabled healthcare services [1], and it is seen as a potential solution to address challenges in healthcare [2]. There is a growing number of eHealth solutions offering innovative services that can potentially complement or replace traditional modes of healthcare delivery. Given that an eHealth solution would impact patients, staff, and organizations, decision-making regarding its adoption needs to be informed by a scientifically rigorous and objective evaluation [3–6]. A case for decision-making regarding investment on an eHealth solution is often based on the results of a summative evaluation. Such an evaluation examines the demonstrated benefits of an eHealth solution in relation to the limited resources [4]. Therefore, there is growing interest in evidence on the efficacy and cost-effectiveness of eHealth [7, 8].

Scholars have argued that scientific rigor is of crucial importance to produce quality evidence in a summative eHealth evaluation [5, 6, 9, 10]. To increase the methodological rigor, continuous learning, and quality of evidence, it is recommended to apply standards such as evaluation frameworks and standard outcome indicators (hereinafter referred to as standards) [5, 6, 11, 12]. Moreover, standardization of eHealth evaluation was recommended in the World Health Organization’s Global eHealth Evaluation Meeting [13], to empower decision-makers with evidence of good quality. In an attempt to create methodological uniformity, and to guide evaluation practitioners with a set procedure and advice, numerous scholars have invested efforts and resources to develop standards for eHealth evaluation planning, execution, reporting and appraisal. With the growing demands for a rigorous approach, several guidelines have been published to assist eHealth evaluators in planning a rigorous eHealth evaluation, such as Health Information Technology Evaluation Toolkit (AHRQ) [14], and Guideline for Good Evaluation Practice in Health Informatics (GEP-HI) [15]. To improve the quality of evidence produced, principles of Health Technology Assessment (HTA) have been considered beneficial to the field of eHealth. HTA is systematic, focuses on the features, effects and consequences of using a technology, and is intended to inform decision making [16]. In addition, multi-disciplinarity, patient-centeredness, and clinical focus makes HTA an attractive methodological source for eHealth evaluation [17]. The principles of the HTA Core Model [18] have been applied in the Model for Assessment of Telemedicine Applications (MAST) [19]. MAST has been tested in multiple pilots covering multiple EU regions, and deemed a beneficial framework in telemedicine studies [20]. However, the MAST model is less suitable to the applications in the early stage of development [21], since the HTA methodology is best suited to collect evidence and inform the decision making regarding the mature technologies. Also, transferability of the economic and organizational evidence is problematic due to strong dependency on the local contexts [20]. Differences in ethical and legal frameworks within the countries can also transferability of the evidence problematic [20]. Such issues need to be considered when applying a standard based on the HTA methodology in a particular situation.

Initiatives such as the Monitoring and Assessment Framework (MAFEIP) [22] developed within the European Innovation Partnership on Active and Healthy Ageing (EIP on AHA) promote the adoption of standards in eHealth evaluation. MAFEIP is a web-based tool that helps calculate health and economic outcomes of social and technological innovations. The estimations in MAFEIP are supported by some specific standards, such as EuroQoL-5D-5 L [23, 24]. The European Commission requests the use of MAFEIP in the calls for research and innovation, promoting the standardized approach to eHealth evaluation. Several scientific journals have also endorsed the use of some standards, such as CONSORT statements for reporting evaluations [25]. CONSORT standards have also been widely accepted in the eHealth community. However, the adoption of other types of standards for conducting an evaluation study, such as eHealth evaluation frameworks and standardized metrics, is currently voluntary. Therefore, it is up to a practitioner’s choice as to how and whether to apply a standard in a particular situation.

Some researchers have suggested that one of the reasons for the lack of generalizable evidence on eHealth outcomes is the insufficient use of standards during evaluation [5, 11, 12, 26]. The use of a standard in a concrete situation in the practical setting can be challenging. For example, eHealth evaluation often involves multiple organizations collaborating on research projects. Interdisciplinary collaborations, such as the Multidisciplinary Translational Teams, can facilitate faster translation of new concepts and findings into real-life improvements in healthcare delivery [27]. Such collaborations can call for an alignment of contested goals and interests [28]. If the standards are considered as a means to increase methodological rigor, to ensure uniformity and continuous learning, and to enable comparability across studies, the usage of standards needs to be understood. However, in the past years, little scholarly attention has been paid to the actual application of standards during an eHealth evaluation.

This study aimed to explore how standards are used in a practical setting of an eHealth evaluation, and to identify the factors that can hinder their use.

## Theoretical framework

To introduce the theory used in this study, it is necessary to briefly explain the research setting (more details are provided in the “Setting” section). The study described in this paper is based on the evaluation planning activities conducted in an eHealth intervention project, implemented by a multi-national and interdisciplinary research consortium. Because of the composition of the consortium and the evaluation planning activities that have been conducted over time, it is considered appropriate to assume an inter-organizational perspective and a process view. As identified in an extensive systematic literature review [29], many studies related to innovation in service organizations, including healthcare, lack process information. The authors advocated for a process perspective in research, as it can help identify potential improvements and enhance innovation processes. Accordingly, to address the purpose of the present study, the process framework of the development of cooperative inter-organizational relationships [30] will be used. The framework is applicable to different kinds of inter-organizational relationships, including a research consortium [30], which makes the framework appropriate for this study.

The process framework for inter-organizational cooperation suggests that cooperation between organizations goes through the sequential phases of negotiation, commitment, and execution. The outcomes of these processes are assessed for efficiency and equity, which may lead to more rounds of the same processes (e.g., re-negotiation). *Negotiations* refer to different explorative activities by participating parties, to develop shared expectations and to clarify each other’s positions, potential resource requirements, and perceived uncertainties of the deal. The *commitments* stage is reached when the parties have achieved a shared agreement that can be fixed informally or formally codified in some way. The agreement can involve commitments, obligations, or other terms for future collaboration. *Executions* occur when the previously achieved agreements are acted upon. *Renegotiations* can occur due to different reasons, such as changes in the situation or positions, or misunderstandings between the parties. After the renegotiation, the terms for collaboration may be changed.

In the present study, the concept of *translation* will be used to understand how the standards are used in a practical setting. Translation can be defined as a process in which one or more actors “tailor the object in such a way that it caters for these people’s explicit interests” [31]. In other words, changes in the original content of the standards can be caused by the agency of the actors. A number of translation studies have been conducted, when standard frameworks were applied in different contexts [32], including healthcare [33]. For example, one study [33] aimed to understand why evidence differs between similar interventions when standard frameworks are applied in a unique context. In that study, local translation was identified as a key factor in determining such differences. To the best of the present author’s knowledge, no study has yet explored how standards are translated in the field of eHealth evaluation.

Four translation strategies have been identified; copying, addition, omission, and alteration [34]. It was indicated that ideas are shaped and modified by using translation strategies during their application in different contexts [32]. *Copying* is the strategy in which the content of an idea is transferred to a particular context, maintaining the original content of the idea. Such strategy is also considered ideal for knowledge transfer. The *addition* strategy indicates that extra elements are added to the original source, in order to match the context. In the *omission* strategy, one removes some elements of the original model, when they cannot be applied to the current context or when the elements do not add value to the situation. The *alteration* strategy is the opposite of copying, as it implies the radical modification of an original model.

A choice of strategies can depend on the content of the original model; the more explicit the original content is, the more likely is the use of the copying strategy [34]. Contrarily, the more abstract the content is, and if the translation concerns different individuals, copying is a less likely strategy. Additionally, the specifics of the field can influence the strategies employed [32, 34].

**Fig. 1:**
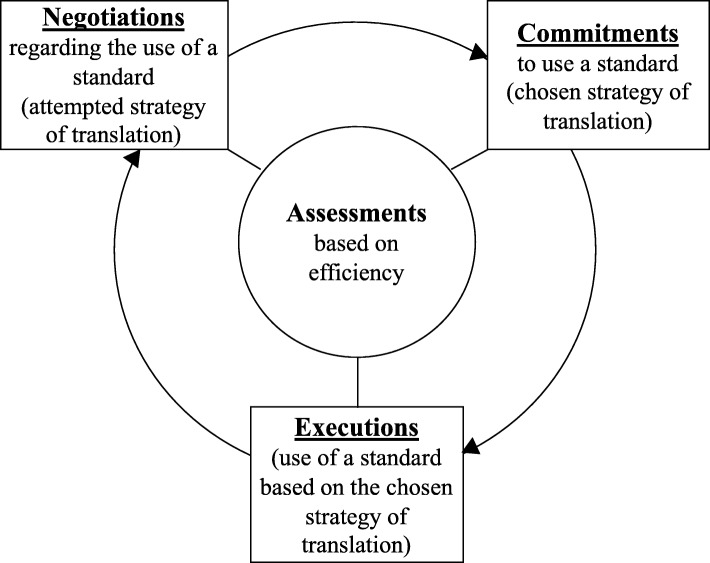
Standards within the process framework of the development of cooperative inter-organizational relationships

The above described theories were used as an analytical framework in the present study, in the following ways. The process framework of the development of cooperative inter-organizational relationships [30] provided a lens to identify the inter-organizational processes that led to the decisions regarding the use of standards in summative eHealth evaluation (see Fig. 1). The barriers to the use of standards will be identified in those processes.

## Methods

### Setting

The study described in this paper was based on the evaluation planning activities conducted in a multi-national and interdisciplinary project “Digital Environment for Cognitive Inclusion” (hereinafter – DECI), funded by the European Union. Eight partners of medical, technological, and scientific backgrounds (some partners had mixed roles) constituted the research consortium. Four hospitals, three research institutions, and one technology company collaborated in the project for 3 years. Every partner was in charge of a different work package in the project. The eHealth solution was applied to elderly individuals with mild cognitive impairment (MCI) and mild dementia (MD) in four heterogeneous healthcare contexts in different countries. It consisted of an integrated care platform, a wearable activity sensor, and web-based cognitive and physical training programs. Evaluation of the study outcomes was one of the tasks in the project. The evaluation planning stage in the project was selected for this study because it involved collaborative decision-making activities within the research consortium, in relation to the standards used during the evaluation. The inclusion of standards in the evaluation methodology of the project was at the center of this study. There was an agreed-upon document of work that formed the basis for all activities in the project. The author of this paper represented one of the scientific partners in the research consortium, and had been involved in the inter-organizational collaboration during the evaluation planning activities.

### Data collection

The contractual scope of the project involved a range of evaluation themes. All the themes were attempted to be measured using a standard. To select the standards for analysis in the present study, relevant minutes from the calls and meetings of the research consortium (*n* = 8) were included in the analysis. Additionally, e-mail documents reflecting correspondence within the consortium during evaluation planning (*n* = 261) were collected.

The following two types of standards were selected for this study: 1) an evaluation framework called Model for Assessment of Telemedicine Applications (MAST) [19] and 2) three standardized metrics that cover several evaluation themes of MAST (quality of life, patient satisfaction, and patient perspectives), namely, the EuroQoL five-dimension questionnaire to assess health-related quality of life (EQ-5D-5 L) [23, 24], Patient Satisfaction Questionnaire (PSQ-18) [35], and Camberwell Assessment of Need for the Elderly – Short Form (CANE-S) [36, 37]. More standards were considered and included in the evaluation methodology of this project. However, a limited number of standards were chosen for the present study. After revision of the data collected, the choice was based on the identification of standards that provide opportunities to capture translation strategies.

### Data analysis

The meeting minutes and e-mail correspondence were examined to identify the conversations and decisions surrounding the selected standards. The data were ordered chronologically using the time stamps of the documents and e-mails. The goal of this activity was to identify the evolution of decisions regarding the use of standards. As a result, a story revealing the evolution of events and decisions around each standard emerged.

Subsequently, the process framework of the development of cooperative inter-organizational relationships [30] was used. The data related to each individual standard were organized as per the elements of the framework. Specifically, the actual events in the project and related quotes from the meeting minutes and e-mail conversations were assigned to the processes of negotiation, re-negotiation, commitment (agreements), and execution. In the data outlined in these processes, additional elements of expectations, uncertainties, and bargaining among the consortium partners were identified. Within these elements, barriers to the use of standards in a practical setting were identified (uncertainties and ideas that demanded bargaining). A formal consent to use the quotes from the conversations was obtained from the authors of the quotes.

In order to understand how the identified factors affected the use of the selected standards and what strategy of translation [34] was chosen, quotations related to, and the decisions taken regarding each standard were analyzed.

## Results

During the eHealth evaluation planning, the use of standards in a practical setting was challenged by various concerns. Through the processes of inter-organizational cooperation, the research consortium aimed to reach consensus in relation to how the selected standards should be applied in the project. A detailed description of the processes has been provided in this chapter. The results have been summarized in Tables 1 and 2.

**Table 1:** Translation of the standards through the process framework of the development of cooperative inter-organizational relationships

Process	MAST	EQ-5D-5 L	PSQ-18	CANE-S
Negotiations	Expectations: - Evaluation domains in the framework should be relevant to the contractual scope Uncertainties: No issues observed Bargaining: No issues observed	Expectations: - Validity in all project locations - Adequacy for the patient group Uncertainties: How should the expectations be aligned when the planned standard is not valid in some project locations? Bargaining: Whether to use a common valid but generic standard or a disease-specific, but different standards for different locations	Expectations: - Validity in all project locations - Adequacy for the patient group Uncertainties: No issues observed Bargaining: - What is the best way to capture patient perceptions?	Expectations: - Validity in all project locations - Adequacy for the patient group Uncertainties: No issues observed Bargaining: - Lack of experience - Human resources needed
Commitments	- Usage of MAST approved standards	- Generic standard EQ-5D-5 L selected	- Mixed method approach selected After re-negotiation, It was decided to only use a qualitative interview	- Qualitative interview approach selected
Executions	Operationalizing evaluation domains of MAST	- Terms for data collection defined	- Terms for data collection defined - Re-negotiated the use of PSQ-18 after doubts emerged - Defining the interview protocol	- Terms for data collection defined - Defining the interview protocol
Translation strategy used	Addition: An extra evaluation domain was added to the MAST domains	Copying: EQ-5D-5 L was applied in its original form	Alteration: Some PSQ-18 elements were converted to questions in a customized qualitative interview	Alteration: Some CANE-S elements were converted to questions in a customized qualitative interview

MAST is a Model for Assessment of Telemedicine Applications [27]; EQ-5D-5 L is a EuroQoL five-dimension questionnaire to assess health-related quality of life [15, 16]; PSQ-18 is a Patient Satisfaction Questionnaire [28]; CANE-S is a Camberwell Assessment of Need for the Elderly – Short Form [29, 30]

**Table 2:** Hindering factors to the use of standards in eHealth evaluation

Hindering factors to the use of standards	
1.	Inadequacy of a standard to address a target population or a disease
2.	Insufficient resources to use a standard
3.	Lack of experience in using a standard
4.	Lack of validation of a standard in a particular context

### Translation of the MAST evaluation framework

Selecting the right framework for an overall evaluation structure was among the first tasks during the evaluation planning. The contractual scope of the project involved the following evaluation themes: quality of life, clinical outcomes, safety, patient and professional satisfaction, technology acceptance, process effectiveness, cost-effectiveness, patient empowerment, and patient value. The MAST framework [19] based on the HTA Core Model [18] was considered for the use in the project. However, professionals' satisfaction was not among the suggested domains of MAST, as requested in the contract of the project. Therefore, this element was *added* to MAST.

No negotiation or bargaining occurred within the research consortium regarding the choice. However, negotiations emerged on a more tangible level when the partners tried to operationalize the individual evaluation domains of MAST, as exemplified in the sections that follow. As evident from this example, the good fit between the evaluation questions outlined in the contract and the conceptual evaluation framework determined the use of a standard. In this case, factors hindering the use of the framework were not observed.

### Translation of the EQ-5D-5 L for measuring quality of life

Quality of life was one of the primary endpoints in the project. Initially, the research consortium considered the standard 12-item Short Form Health Survey SF-12 [38] as general health-related quality of life indicator. Another possible indicator was Quality of Life in Alzheimer’s Disease (QoL-AD) [39] which is appropriate for use with individuals with mild cognitive impairment and mild dementia. This standard had also been applied in a similar study [40]. However, three out of the four clinical partners from different countries found that the QoL-AD did not have a validated local language version. The same reason determined the non-use of SF-12.

Extract 1 (from an e-mail sent by a clinical neuropsychologist (male, Italy, 14 years of professional experience) to the research consortium):

“… QoL-AD /…/ have no /language/ translation and validation. /…/ also for /country/ and /country/. We think that it could be risky, from a methodological point of view, to not have a sound and validated measure for a primary endpoint.”

The research consortium was uncertain about the methodological risk of using a non-validated tool for a primary endpoint. Subsequently, a round of negotiations emerged, which were related to a trade-off between adequacy of the standard to the patient group and availability of local translations and validations. Then, the partners discussed potential implications of the alternative solutions.

Extract 2 (from an e-mail sent by a clinical neuropsychologist (male, Italy, 14 years of professional experience) to the research consortium):

“… If there is not a validated single version of a tool for each cultural context, we suggest to use different validated tools for the same variable and discussing how to compare the data among the clinical sites.”

However, the use of non-validated or different questionnaires among the partners for the same variable was not desirable, as it would complicate the cross-country analysis during the evaluation. When a decision could not be reached easily, an extra insight was shared by another partner with higher competence in health economic evaluation, which was an additional evaluation theme in the project. The partner suggested the use of the standard EQ-5D-5 L [23, 24] for quality of life measurement instead, as it would be more beneficial for the cost-benefit analysis in the MAFEIP [22]. The EQ-5D-5 L is a generic measure of quality of life that can be applied in a broad array of healthcare studies. Translations into relevant local languages were found to be validated for all contexts of the partners involved. The extra utility of the EQ-5D-5 L for the cost-benefit analysis helped reach a decision, and the negotiations stopped. As the commitment to use EQ-5D-5 L was established, the decision was documented in the evaluation plan, along with other details regarding data collection.

As seen from this example, the unavailability of a translated and validated standard in all involved research contexts hindered the use of a disease-specific standard, the QoL-AD. As the EQ-5D-5 L was not modified, and was used in its original form, the *copying* strategy was applied in this case.

### Translation of the PSQ-18 questionnaire for measuring patient satisfaction

The measure proposed for assessing patient satisfaction was the generic eighteen-item Patient Satisfaction Questionnaire (PSQ-18) [35]. When aiming to decide on a common standard for measurement, the first round of negotiations among the partners concerned differences regarding the preferred methodological approach. While some partners argued for a quantitative approach using the PSQ-18 alone, others preferred a mixed-method approach by combining the PSQ-18 with a qualitative interview. However, during negotiations, it was perceived that the PSQ-18, being a quantitative measure, lacks the opportunity to capture patients’ perceptions in their own language. Then, all partners agreed upon a decision to use the PSQ-18 along with a semi-structured interview. As the commitment was established, the decision was documented in the evaluation plan.

After having applied the PSQ-18 with the first set of patients, one clinical partner expressed doubts about its usefulness for patients with cognitive impairment. The partner emphasized that some questions were difficult for such patients to comprehend, at the same time causing extra cognitive load. These insights triggered a round of re-negotiation that questioned the necessity and value of a quantitative measure when the patients had a limited capability to comprehend the questions.

Extract 3 (from an e-mail sent by a researcher (female, Sweden, 2 years of professional experience) to the research consortium):

“… After data collection started and first experiences came, clinical partners commonly agreed that the PSQ-18 is not a suitable tool for satisfaction measurement. Therefore, we will rely on the interview questions, which will ask patients for their feedback regarding the key areas of intervention or regular care.”

It was decided to create interview questions that build on a limited range of topics from the PSQ-18 (8 instead of 18 questions in the PSQ-18). The questions were to be asked to a limited sample of patients. The agreed-upon interview questions and the interview sampling procedures were documented in the evaluation plan.

As seen from this example, the issues related to the adequacy of the standard for the target patient group led the research consortium to decide to discontinue the use of this standard in its original form. The standard PSQ-18 was therefore *altered* into a custom solution, in the form of a qualitative interview. Several elements of the PSQ-18 were converted into interview questions.

### Translation of the CANE-S for elderly needs assessment

The CANE-S [36, 37] is a standard dedicated to the assessment of the service compatibility with the needs of elderly people with mental disorders. It is used in research to describe the met and unmet needs of such patients (e.g., [41]). Therefore, the CANE-S was considered to be included in the evaluation plan of the project, to address the evaluation domain of patient value. However, different views of the partners emerged.

Extract 4 (from an e-mail sent by a quality director (male, Sweden, 27 years of professional experience) to the research consortium):

“… the CANE-S is available in /country/ translation; though, at the moment, we have not confirmed whether it is equal in all respects to the English version. Furthermore, the CANE-S is not in regular use in /country/, neither in /hospital/. /…/ teaching how to use a brand new instrument must be judged as an insurmountable task for the time schedule at hand.”

Extract 5 (from an e-mail sent by a researcher (male, Spain, 8 years of professional experience) to the research consortium):

“… We have observed a disagreement in the use of the CANE-S. /…/ We do not have experience in administering that test, so consequently, we cannot provide a strong opinion about using it or not (we know that is quite time consuming, but probably very useful).”

Inclusion of the CANE-S was advocated by the partners who had experience in using the standard. The disadvantages of using it were mainly raised by the partners who had not used it in their regular practice. A concern regarding the resources needed to administer the standard was also raised, considering the several other measures that were to be used during the evaluation. The final decision was to remove the CANE-S from the scope of evaluation. Instead, it was decided to cover the service compatibility with patient needs partially through the qualitative interviews. The agreement was documented in the meeting minutes.

As seen from this example, factors such as a lack of experience and resources needed to use a standard led to the *alteration* of the CANE-S. A custom solution was created in the form of a qualitative interview. Several elements of the CANE-S were converted into interview questions.

Table 1 provides a summary of the results in relation to each standard analyzed.

Table 2 provides a list of the hindering factors related to the use of standards.

## Discussion

The present study aimed to explore how standards are used in a practical setting of an eHealth evaluation, and to identify the factors that can hinder their use. Different initiatives to standardize eHealth evaluation (e.g. MAFEIP [22], MAST [19]) provide a blueprint for the process and topics to evaluate, but they do not consider the practicalities of such work. This study demonstrated that implementing such frameworks is problematic and the use of standards differs considerably. The different translation strategies [34] employed in the case studied denote the varying extents of the use of standards as well as the variations in adherence to their original content. The results indicate that, if a particular standard was used in different evaluation studies, different levels of adherence can create variance in evaluation methodologies and can affect the generalizability of the evidence generated. However, the translation strategies can increase the quality of the evaluation study in terms of a better fit between the standards and the circumstances. These findings add knowledge on the view that the application of standards leads to uniformity among different studies [5, 11, 12, 26].

The results also expand on the previously identified complexities in eHealth evaluation emerging from social contexts when multiple organizations collaborate on research projects [27]. In addition to the previously identified needs to align contested goals and interests, the present study added the hindering factors related to the use of standards. The factors concern lack of experience in using a standard, insufficient resources to use a standard, lack of validation of a standard in a particular location, and inadequacy of a standard to address a target population or a disease.

The following theoretical contributions can be identified from the present study. First, the use of a standard needs to be understood as a range, and not a scale (i.e., whether a standard is used or not), as presented in previous research [5, 6, 11, 26]. In reality, the standards may be modified to increase their fit to the practical circumstances. However, it could lead to a varying range of adherence. Consequently, it affects the generalizability of the evidence generated.

The second theoretical contribution of this study is the finding that the use of standards may be affected by the social processes involving different perspectives and needs of the collaborating actors. It adds complexity to the choice of standards and can hinder adherence to their content. Hence, existence of multiple collaborating actors can produce heterogeneity in the evaluation methodologies and generated evidence.

A number of practical recommendations can be identified from this study. Social complexity needs to be taken into account during decision making regarding the use of standards in a multi-party eHealth evaluation. It may be time-consuming to resolve the barriers and to reach a consensus regarding the use of standards.

Further, the use of standards can be enhanced if a higher number of standards is translated and validated in different geographical locations and for a variety of populations. Availability of a valid local version applicable to a particular population can stimulate the use of a standard and can ensure continuous learning and methodological uniformity among different studies. It can also facilitate multi-national eHealth evaluations, as the decision regarding the use of the same standard in different locations would not be hindered by the unavailability of valid local versions.

### Limitations and future research

The factors identified in this study may possibly not be final due to the limited number of standards included in this study. Additionally, the case study design employed in this study delimited findings because a single research setting was studied. The main data were collected from e-mail correspondence between the partners in a research consortium. Other means of data collection were not explored in this study. A limited number of standards (evaluation frameworks and outcome measures) were introduced to the research consortium due to the practical constraints of the project. The circumstances delimited the time for exploration of the optimal standards and learning how to use them. Therefore, selection of the standards used in the evaluation relied on the regular measurement practices in the project locations.

Future research should aim to identify other possible factors limiting the use of standards in different settings of eHealth evaluation. Additionally, mechanisms to enhance the use of standards need to be explored.

## Conclusions

During decision making regarding the use of standards, collaborative processes differ based on the type of a standard. For evaluation frameworks that are more conceptual and abstract than standardized metrics, the decision to use the framework can easily be reached as long as it fits purposes of the project. On the contrary, when decisions concern standardized metrics that are more tangible and require the actual work of evaluators, negotiations emerge.

Further, the following factors were identified as barriers to the use of standards during evaluation: (1) inadequacy of a standard to address a target population or a disease, (2) insufficient resources to use a standard, (3) lack of experience in using a standard, (4) lack of validation of a standard in a particular context. These factors can hinder adherence to the standards and cause changes in their original versions. Hence, even if standards are used in eHealth evaluation studies, practical circumstances and the existence of multiple collaborating actors can produce heterogeneity in the evaluation methodologies and can affect the evidence generated.

## Data Availability

The datasets used and/or analyzed during the current study are not publicly available due to the participant confidentiality reasons, but are available from the corresponding author on reasonable request.

## Abbreviations

CANE-S: Camberwell Assessment of Need for the Elderly—Short Form [36, 37]
CONSORT: Consolidated Standards of Reporting Trials [25]
EQ-5D-5L: EuroQOL five-dimensional Level 5 questionnaire [23, 24]
HTA: Health Technology Assessment
MAFEIP: Monitoring and Assessment Framework for the European Innovation Partnership on Active and Healthy Ageing [22]
MAST: Model for Assessment of Telemedicine Applications [19]
PSQ-18: Patient Satisfaction Questionnaire [35]
QoL-AD: Quality of Life in Alzheimer’s Disease [39]
SF-12: Short Form Health Survey [38]
